# Ectopic expression of Snord115 in choroid plexus interferes with editing but not splicing of 5-Ht2c receptor pre-mRNA in mice

**DOI:** 10.1038/s41598-019-39940-6

**Published:** 2019-03-12

**Authors:** Carsten A. Raabe, Reinhard Voss, Delf-Magnus Kummerfeld, Juergen Brosius, Chenna R. Galiveti, Anna Wolters, Jochen Seggewiss, Andreas Huge, Boris V. Skryabin, Timofey S. Rozhdestvensky

**Affiliations:** 1 Institute of Experimental Pathology (ZMBE), University of Muenster, Von-Esmarch-Str. 56, D-48149 Muenster, Germany; 2 Institute of Medical Biochemistry (ZMBE), University of Muenster, Von-Esmarch-Str. 56, D-48149 Muenster, Germany; 3 Laukamp 12, D-48161 Muenster, Germany; 4 Medical Faculty, Core Facility Transgenic animal and genetic engineering Models (TRAM), University of Muenster, Von-Esmarch-Str. 56, D-48149 Muenster, Germany; 5 Brandenburg Medical School (MHB), Fehrbellinerstr. 38, D-16816 Neuruppin, Germany; 6 Institute of Human Genetics, University Hospital Muenster, Vesaliusweg 12-14, D-48149 Muenster, Germany; 7 Medical Faculty, Core Facility Genomik, University of Muenster, Albert-Schweitzer-Campus 1, D3, Domagkstrasse 3, D-48149 Muenster, Germany

## Abstract

Serotonin 5-HT2C receptor is a G-protein coupled excitatory receptor that regulates several biochemical pathways and has been implicated in obesity, mental state, sleep cycles, autism, neuropsychiatric disorders and neurodegenerative diseases. The activity of 5-HT2CR is regulated *via* alternative splicing and A to I editing of exon Vb of its pre-mRNA. Snord115 is a small nucleolar RNA that is expressed in mouse neurons and displays an 18-nucleotide base complementary to exon Vb of 5-HT2CR pre-mRNA. For almost two decades this putative guide element of Snord115 has wandered like a ghost through the literature in attempts to elucidate the biological significance of this complementarity. In mice, Snord115 is expressed in neurons and absent in the choroid plexus where, in contrast, 5-Ht2cr mRNA is highly abundant. Here we report the analysis of 5-Ht2cr pre-mRNA posttranscriptional processing *via* RNA deep sequencing in a mouse model that ectopically expresses Snord115 in the choroid plexus. In contrast to previous reports, our analysis demonstrated that Snord115 does not control alternative splicing of 5-Ht2cr pre-mRNA *in vivo*. We identified a modest, yet statistically significant reduction of 5-Ht2cr pre-mRNA A to I editing at the major A, B, C and D sites. We suggest that Snord115 and exon Vb of 5Ht2cr pre-mRNA form a double-stranded structure that is subject to ADAR-mediated A to I editing. To the best of our knowledge, this is the first comprehensive Snord115 gain-of-function analysis based on *in vivo* mouse models.

## Introduction

The 5-hydroxytryptamine receptor 2C (5-HT2CR) belongs to the superfamily of G-protein coupled receptors and interacts with its endogenous ligand, the neurotransmitter serotonin (5-hydroxytryptamine, 5-HT). 5-HT2C receptor signaling regulates a wide variety of biochemical circuits, which among others, have been implicated in obesity, appetite, mental state, sleep cycles, autism, neuropsychiatric disorders (e.g., schizophrenia, depression) and neurodegenerative diseases (e.g., Parkinson, Alzheimer)^1–4^.

**Figure 1:**
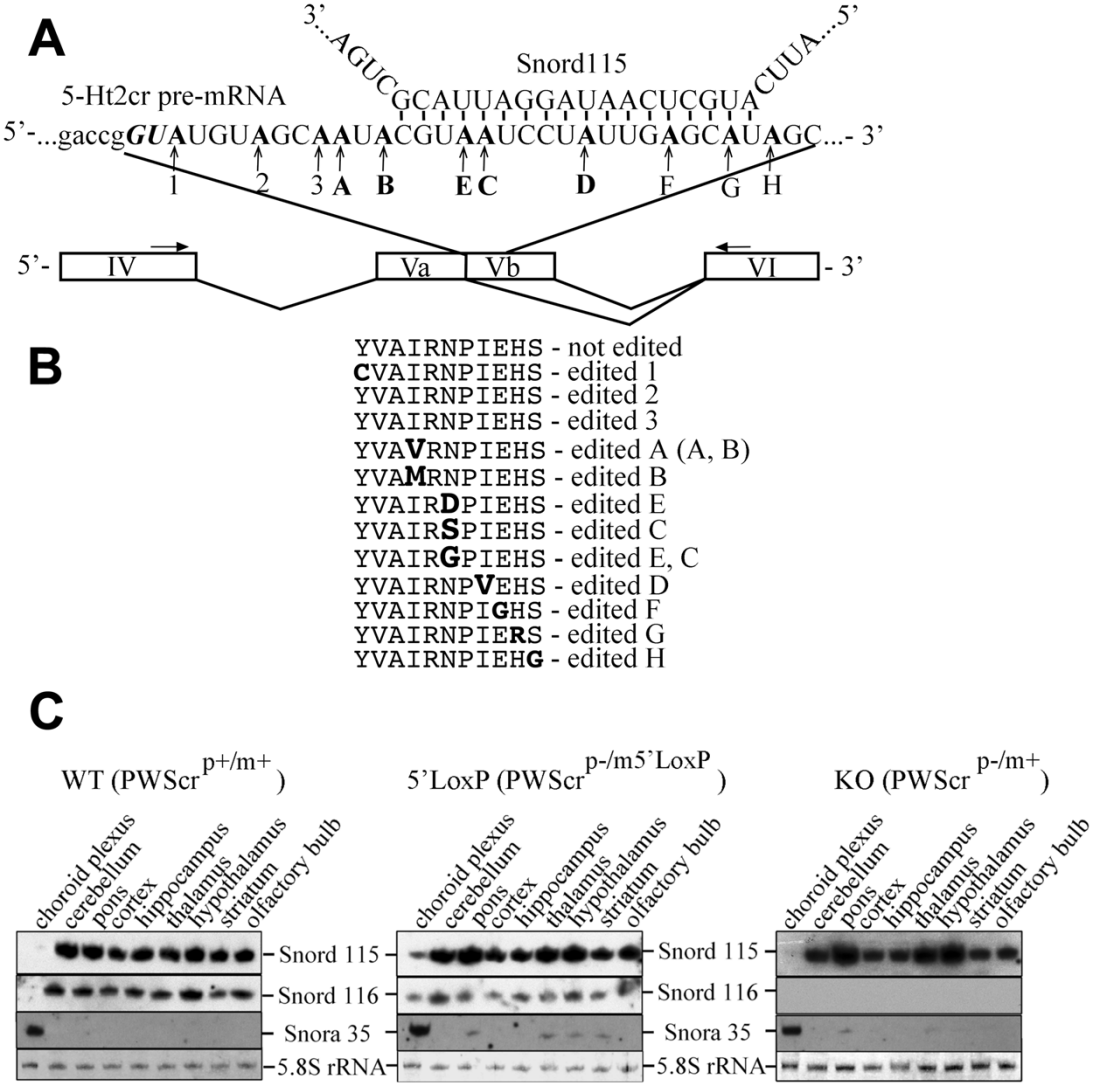
Schematic representation of putative 5-Ht2cr pre-mRNA targeting by Snord115 and snoRNA expression in different brain areas. (**A**) Putative base pairing of Snord115 with exon Vb of 5-HT2cr pre-mRNA (upper panel). The alternative splice site (*GU*) of exon Vb and A to I editing sites in 5-Ht2cr pre-mRNA (1-H) are indicated. Schematic representation of 5-Ht2cr pre-mRNA exons IV-VI is shown in the lower panel. Black arrows indicate the primer positions. (**B**) Amino acid variants of 5-Ht2c receptor encoded by different mRNA editing isoforms. (**C**) Northern blot analysis of total RNA isolated from different brain areas (indicated above blots) of wild type, *PWScr*^*p*−*/m5′LoxP*^ and *PWScr*^*p*−/*m*+^ mice. Bands of Snord115, Snord116 and Snora35 RNAs are indicated. As loading control, 5.8S rRNA (negative image of an ethidium bromide stained gel) is shown at the bottom.

In human and mouse, the 5-HT2CR receptor is particularly abundant within epithelial cells of the choroid plexus^5,6^. Alternative splicing of exon V of the receptor hnRNA (heterogeneous nuclear RNA) leads to a truncated receptor variant (5-HT2CR-tr), which encodes a non-functional 5-HT2CR isoform lacking the coding sequences for the second internal loop and parts of the fourth transmembrane domain (Fig. 1A,B).

The efficiency of 5-HT2CR G-protein coupling is regulated by posttranscriptional A to I editing within the CDS (coding sequence) of the second internal loop of the receptor^7–9^. ADAR1 and ADAR2 (adenosine deaminase acting on RNA) enzymes catalyze receptor pre-mRNA (precursor mRNA) editing at five different sites: ADAR1: A, B, C and E – sites and ADAR2: C, D and E sites, respectively^10–13^. Combinations of these individual editing events amount to 32 different 5-HT2CR mRNA isoforms, which collectively modulate the serotonergic signal transduction to varying degrees^14,15^.

The imprinted SNORD116 and SNORD115 (formerly designated as HBII-85 and HBII-52, respectively) are small nucleolar RNAs (snoRNAs), each arranged in gene clusters in the Prader-Willi syndrome locus (PWS)^16^. The RNAs harbor conserved C and D box sequence motifs and belong to the subclass of 2′-O-methylation guide C/D box snoRNAs. Thus far, no significant base complementarities with “classical” snoRNA targets such as ribosomal RNAs (rRNAs) have been established; therefore, they are categorized as “orphan” snoRNA with potentially non-canonical functions^16,17^.

The neurogenetic disease PWS manifests mainly as a result of large genomic deletions on human chromosome 15 involving the snoRNA clusters, including its non-protein coding host RNA, and several protein coding genes^18^. Analyses of mouse models that lack the Snord116 genomic cluster suggest a critical region associated with PWS (*PWScr*)^19,20^. Subsequently, several patients with corresponding deletions, involving only the *SNORD116* cluster, were reported^21–23^. Mouse models harboring paternal deletions of the Snord116 cluster exhibit postnatal growth retardation and, depending on the mouse background strain, up to 15% of postnatal lethality^19,20^. A potential involvement of SNORD115 in PWS, however, remains elusive, as loss of the SNORD115 cluster does not contribute to the PWS phenotype in humans^24^.

Canonical C/D box RNAs guide 2′-O-methylation of target RNAs. Base pairing interactions of snoRNAs with their molecular targets, i.e., rRNAs and snRNAs (small nuclear RNAs) select the corresponding nucleotides for ribose modification^25,26^. The antisense element of SNORD115 displays conserved base complementarities of 18nt to the alternatively spliced 5-HT2CR pre-mRNA exon Vb (Fig. 1A)^16,17,27,28^. This region is also subject to posttranscriptional A to I editing; the potential of the SNORD115 guide element to regulate A to I editing was demonstrated with artificial RNAs in cell culture experiments^13^.

Alternative splicing resulting in a non-functional truncated serotonin receptor variant, depends on a splice site, which is located in close proximity (13nt upstream) to the region of this hypothetical snoRNA/hnRNA interaction. Indeed, SNORD115 has been reported to interfere with alternative splicing of the 5-HT2CR pre-mRNA *in vitro* and *ex vivo*^29,30^.

Because of the complementarity of the Snord115 guide element to a crucial region of the 5-Ht2c receptor pre-mRNA, most research focused on this potential interaction. Thus far, the *in vivo* analysis of differences in RNA editing for 5-Ht2c receptor pre-mRNA in mouse models revealed controversial results and lacked sufficient sequencing depth^31–33^. Here, we report RNA deep sequencing analyses of 5-Ht2cr pre-mRNA posttranscriptional processing in wild type and a mouse model^34^ that constitutively expresses Snord115 in choroid plexus.

## Results

### Mouse lines and experimental design

We recently generated a knock-in (KI) mouse model containing a 5′ HPRT-LoxP-Neo^R^ cassette (5′LoxP) inserted upstream of the *Snord116* and *Snord115* snoRNA gene clusters^34^. Heterozygous KI female mice that harbored the modified maternal allele (*PWScr*^*p*+/*m5*′*LoxP*^) were crossed with Snord116 knock-out (KO) mice (*PWScr*^*p*−*/m*+^) that lack the snoRNA cluster within the transcriptionally active paternal gene locus^20^. The resulting offspring displayed *PWScr*^*p*−*/m*+^, *PWScr*^*p*−*/m5*′*LoxP*^ and wild type genotypes.

In contrast to wild type and *PWScr*^*p*−*/m*+^ mice, the *Snord116*^*P*−*/m5*′*LoxP*^ animals express Snord116 and Snord115 snoRNAs within the choroid plexus (besides several non-brain tissues) (Fig. 1C)^34^. With RNA high throughput sequencing, we analyzed the patterns of 5-Ht2cr pre-mRNA posttranscriptional processing in *PWScr*^*p*−*/m5*′*LoxP*^ and wild type mice. This *in vivo* experimental design enabled—for the first time—the identification of potential interactions of Snord115 with 5-Ht2cr pre-mRNA. In particular, differences in alternative splicing and/or RNA editing of the serotonin receptor pre-mRNA in the choroid plexus of *PWScr*^*p*−*/m5*′*LoxP*^ and wild type animals (*PWScr*^*p*−*/m5*′*LoxP*^ - LoxP and wild type - WT) were investigated.

Twelve male mice pooled in six biological replicates (two choroid plexuses per sample) for each genotype were analyzed. Oligonucleotides specific for exon IV and VI (Fig. 1A) enabled the RT-PCR amplification of receptor pre-mRNA using total RNA. The PCR products were processed on an Ion PGM sequencing platform for RNA deep sequencing.

### Snord115 expression in choroid plexus and 5-Ht2cr pre-mRNA alternative splicing

The resulting cDNA reads were mapped against the two splice variants of the 5-Ht2cr mRNA, i.e., a longer isoform of 209nt that contained the A to I editing sites (5-Ht2cr) and a shorter variant of 114nt, representing the truncated isoform of the serotonin receptor (5-Ht2cr-tr). Analysis of RNA expression to quantify the relative abundance of both 5-Ht2cr/5-Ht2cr-tr mRNA variants for wild type and *PWScr*^*p*−*/m5*′*LoxP*^ samples revealed no significant difference with ~55% and ~45% of all cDNA reads representing the truncated and functional receptor isoforms, respectively (Table 1., Supplementary Table 1). Our data indicate that the heterologous expression of Snord115 RNA within the choroid plexus of *PWScr*^*p*−*/m5*′*LoxP*^ mice had no impact on 5-Ht2cr pre-mRNA alternative splicing *in vivo* (Table 1).

**Table 1:** Average number of reads for 5-Ht2cr alternative splicing variants within exon V of pre-mRNA in choroid plexus of *PWScr*^*p*−*/m5′LoxP*^ and wild type male mice.

Genotype	Average mapped reads	Average 5-Ht2cr reads	Average 5-Ht2cr-tr reads	5-Ht2cr reads %	5-Ht2cr-tr reads %
*PWScr* ^*p*− */m5′loxP*^	6493463	2942551	3550911	~45,3%	~54,7%
WT	5569933	2516839	3053094	~45,2%	~54.8%

*Genotype*: Genotypes of investigated mice. *Average mapped reads*: Average number of mapped reads of all BAM files. *Average 5-Ht2cr reads*: Average number of reads obtained for full-length 5-Ht2cr mRNA. *Average 5-Ht2cr-tr reads*: Average number of reads obtained for truncated (alternatively-spliced) 5-Ht2cr-tr mRNA. *5-Ht2cr reads and 5-Ht2cr-tr reads %*: The percentage of all cDNA reads representing the functional and truncated receptor isoform is indicated.

### ADAR expression does not change in different genotypes

ADAR1 and ADAR2 enzymes catalyze A to I editing within exon Vb of the receptor pre-mRNA. Here, the enzymes recognize double-stranded RNA structures that are formed between intron V and exon Vb of 5-Ht2cr pre-mRNA (Supplementary Fig. 1)^28^. To exclude the remote possibility that ADAR expression differs substantially between *PWScr*^*p*−*/m5*′*LoxP*^ and wild type siblings, we analyzed ADAR expression with quantitative real-time PCR (RT-qPCR). Three brain samples from postnatal day 21 male mice were collected per each investigated genotype. This allowed the comparison of *PWScr*^*p*−*/m5*′*LoxP*^ and wild type mice of the same litters for all three samples. RT-qPCR analysis revealed that expression levels for ADAR1 and ADAR2 were unaltered (Supplementary Table 2A). We collected four *PWScr*^*p*−*/m5*′*LoxP*^ and six wild type samples from male siblings at postnatal day P21 for the RT-qPCR analysis of *ADAR1* and *ADAR2* expression levels in choroid plexus. Our data demonstrate that in choroid plexus similar to the whole brain, ADAR1 and ADAR2 mRNA levels were the same for *PWScr*^*p*−*/m5*′*LoxP*^ and wild type mice (Supplementary Table 2B).

### Snord115 expression in choroid plexus and A to I editing

To date, five different 5-Ht2cr mRNA editing sites (A, B, E, C and D) all of them located within an 20nt stretch of exon Vb are known (Fig. 1, Supplementary Fig. 1)^30^. All combinations of these five different editing sites amount to 32 mRNA isoforms and 24 different 5-Ht2cr-protein variants presented in Fig. 1A,B. We extended this original analysis of the Vb exonic region to 30 nucleotides and monitored RNA editing at six additional positions designated as 1, 2, 3, F, G and H (Fig. 1A,B). Hence, eleven sites and their combinations were examined to identify their contributions to RNA editing. Our experimental design also enabled analysis of coupled editing events apart from RNA editing at single sites. Resulting combinations were labeled according to the contributing individual editing sites (e.g., 123 A, ABD, etc.); cDNA reads representing the non-edited isoform were labeled ‘NoEdit’. For comparison of individual variants and their contribution to RNA editing across samples, we normalized isoform expression by the contig length and the weight of the experiment. The resulting RPKM (Reads Per Kilobase of transcript, per Million mapped reads) values enabled the analysis of differential expression between genotypes and, in addition, to rank the relative abundance of isoforms within samples. Only isoforms with mean expression threshold levels of ≥10 RPKM were further analyzed (Materials and Methods) (Supplementary Table 3).

### RNA editing levels differed between wild type and LoxP mice

In order to analyze and detect overall differences in RNA isoform expression between wild type and *PWScr*^*p*−*/m5*′*LoxP*^ samples, we calculated the mean expression representing the average of all edited and non-edited isoforms across all six biological replicates for each genotype separately. The resulting expression values, i.e., RPKM, represent a direct, normalized measure of RNA editing levels per sample. These values were further evaluated *via* t-tests for identification of statistically relevant differences. Overall, this analysis revealed only a small decrease (~5%) of 5-Ht2cr mRNA editing in the choroid plexuses of *PWScr*^*p*−*/m5*′*LoxP*^ mice (64%) compared to wild type (69%) (Supplementary Table 3). This suggested that within our *in vivo* system, snoRNA115 might contribute to the fine-tuning of A to I editing.

### Analysis of differential expression of RNA editing isoforms

To identify A to I editing isoforms that were differentially expressed in *PWScr*^*p*−*/m5*′*LoxP*^ and wild type samples during analysis of decreased editing levels in *PWScr*^*p*−*/m5*′*LoxP*^ mice, the expression of 112 editing variants (i.e., including the non-edited isoform) was investigated across six biological replicates for each genotype *via* t-tests. All p-values were corrected for multiple testing.

We identified a total of 111 different 5-Ht2cr pre-mRNA editing isoforms. Only 26 individual variants accounted for ≥0,1% abundance, whereas ~50 isoforms were detected at ≥0.01% (Supplementary Table 3). The most prevalent isoform was the single D-site edited 5-Ht2cr pre-mRNA representing ~61% of all edited transcripts in the wild type choroid plexus samples. Most edited isoforms that were present at ≥0,1% contained different combinations of the five previously reported A, B, E, C and D editing sites. Also, the 2D and DG editing isoforms in wild type and 2D, DG, 1D and DH in *PWScr*^*p*−*/m5*′*LoxP*^ mice constituted ≥0.1% (Supplementary Table 3). With the analysis of differential expression for all 5-Ht2cr pre-mRNA editing variants across samples and t-tests, we detected significant changes of individual isoform expression. Statistically significant differences between *PWScr*^*p*−*/m5*′*LoxP*^ and wild type 5-Ht2cr pre-mRNA editing isoforms were observed for 21 variants (Table 2). Ten of these variants account for more than 0.1% of the total edited transcripts. Most differences in editing were observed for BCD, AECD, ABCD, ABD and AC 5-Ht2cr pre-mRNA isoforms, revealing 4.9, 2.8, 2.5, 1.75 and 1.6-fold editing decrease, respectively, when *PWScr*^*p*−*/m5*′*LoxP*^ choroid plexus samples were compared to wild type (Table 2). When the contributions of single sites to detected editing patterns were analyzed, we observed that D-site editing is prevalent (Table 3). When editing patterns of 5-Ht2cr mRNA in wild type mouse choroid plexus were analyzed, we detected that the D-site was involved in nearly 89.5% of all editing followed by contributions of C- (23.2%), A- (14.7%), B- (6.5%) and E-sites (5.1%). Other editing sites contributed less then 1% to identified 5-Ht2cr pre-mRNA editing patterns. Single-site changes in editing of 5-Ht2cr pre-mRNA between analyzed mouse genotypes are summarized in Table 3. Significant, albeit small, differences in RPKM values between *PWScr*^*p*−*/m5*′*LoxP*^ and wild type mouse choroid plexus samples were detected for A, B, C and D editing sites (Table 3). Editing at other sites did not contribute statistically significant differences in 5-Ht2cr pre-mRNA editing. In summary, our data indicate that the presence of Snord115 in choroid plexus has a moderate, yet statistically significant influence on 5-Ht2c-receptor pre-mRNA editing—increasing the ratio of non-edited isoforms *in vivo*.

**Table 2:** Significant changes of 5-Ht2cr mRNA editing isoforms between *PWScr*^*p*−*/m5*′*LoxP*^ and wild type mice choroid plexus samples.

Pattern	Mean RPKM WT	Mean RPKM LoxP	Fold change WT/LoxP	P-value	SIG	pBH	WT %	LoxP %
CD	126959	104482	1.2	0.0009	***	0.02	12.6	11.8
AD	44104.5	33894	1.3	0.0013	**	0.0185	4.4	3.8
ABD	32223	18402	1.75	6.52E-05	****	0.0072	3.2	2.1
ED	23259	19153	1.2	0.03068	*	0.179	2.3	2.17
ACD	19010	14490	1.3	0.0043	**	0.053	1.9	1.6
ABCD	12761	5132	2.5	0.00027	***	0.0099	1.3	0.58
E	11309	17713	0.64	0.0062	**	0.0627	1.1	2
AC	5546	3450	1.6	0.00095	***	0.0175	0.55	0.3
AECD	3544	1277	2.8	0.0098	**	0.09	0.35	0.14
BCD	2631	535	4.9	0.024	*	0.156	0.26	0.06
CDH	262	79	3.3	0.019	*	0.1288	0.026	0.009
1AD	64.5	44	1.46	0.037	*	0.1973	0.006	0.005
ADG	61.5	46	1.34	0.044	*	0.2218	0.006	0.0052
ABDG	44	25	1.8	0.029	*	0.1792	0.004	0.0028
1ABD	35.5	22	1.6	0.012	*	0.0867	0.0035	0.0025
ABCDG	24.6	8	3.2	0.0013	**	0.0185	0.002	0.00087
ABCDF	21	6.5	3.2	0.00016	***	0.009	0.002	0.0007
3ABCD	19.5	7	2.9	0.005	**	0.0554	0.0019	0.00076
1ABCD	19	4	5	0.0003	***	0.0087	0.00185	0.0004
ABCDH	12	3	3.6	0.0112	*	0.0867	0.0012	0.0004
3AC	11	5	2.33	0.01	*	0.0867	0.001	0.00056

*Pattern*: Combinations of edited sites contribute to a variety 5-Ht2cr mRNA editing patterns. They are labeled according the designations of the contributing editing sites (Fig. 1A). *Mean RPKM WT and LoxP*: Calculated mean of RPKM values for wild type (WT) and, *PWScr*^*p*−*/m5*′*LoxP*^ (LoxP) samples. *Fold change WT/LoxP:* Fold change between wild type (WT) and *PWScr*^*p*−*/m5*′*LoxP*^ (LoxP) editing patterns. *P-value*: P-value of Student’s t-test. *SIG*: Graphpad formatting - asterisks describe p-values levels of statistical significance. *pBH*: “False Discovery Rate” correction for multiple comparisons. WT(%) and LoxP(%): The percentage of detected receptor isoforms in wild type (WT) and *PWScr*^*p*−*/m5*′*LoxP*^ (LoxP) mouse choroid plexuses, is indicated.

**Table 3:** Single editing site contribution to changes in editing patterns of different 5-Ht2cr pre-mRNA isoforms between *PWScr*^*p*−*/m5*′*LoxP*^ and wild type mice choroid plexus samples.

Edited Site	Mean RPKM WT	Mean RPKM LoxP	Fold change (WT/LoxP)	P-value, SIG	WT, %	LoxP, %
1	2275	2225	1	0.67, no	0.2	0.2
2	4129	3295	1.3	0.14, no	0.4	0.4
3	2119	2078	1	0.8, no	0.2	0.2
A	148907	100311	1.5	0.000057^****^	14.7	11.3
B	66074	37479	1.8	0.0003^***^	6.5	4.2
C	234736	190987	1.2	0.0004^***^	23.2	21.6
D	904161	782103	1.2	0.006^**^	89.5	88.4
E	51944	45946	1.1	0.15, no	5.1	5.2
F	1877	1891	1	0.94, no	0.2	0.2
G	3242	2378	1.4	0.14, no	0.3	0.23
H	1940	1878	1	0.84, no	0.2	0.26

*Editing site*: The editing sites are labeled according to Fig. 1A. *Mean RPKM WT and LoxP*: Calculated mean of all RPKM values for 5-Ht2cr mRNA editing patterns accounting for site contribution in wild type (WT) and, *PWScr*^*p*−*/m5*′*LoxP*^ (LoxP) editing. *Fold change WT/LoxP:* Fold change between wild type (WT) and *PWScr*^*p*−*/m5*′*LoxP*^ (LoxP) editing sites. *P-value*: P-value of Student’s t-test. *SIG*: Graphpad formatting - asterisks describe p-values levels of statistical significance. False Discovery Rate (pBH) values were calculated for the correction of multiple testing; pBH values 0.0006, 0.001, 0.0016 and 0.016 for editing sites A, B, C and D, respectively, confirmed significances of the t-test. WT(%) and LoxP(%): The percentage of detected editing in the depicted sites in wild type (WT) and *PWScr*^*p*−*/m5′LoxP*^ (LoxP) mouse choroid plexuses is indicated. Percentage is calculated based on the mean RPKM values for 5-Ht2cr mRNA-edited isoforms in investigated genotypes.

An interesting observation came from the RNA deep sequencing analysis of small non-protein coding RNAs isolated from wild type mouse brain (Rozhdestvensky *et al*. unpublished). We identified that approximately 3% of Snord115 cDNAs represented putatively edited snoRNA isoforms (Supplementary Fig. 1). Notably, A to I editing was specifically detected at residues located within the Snord115 targeting region, which displays base complementarities to exon Vb of 5-Ht2cr pre-mRNA. *PWScr*^*p*−*/m5*′*LoxP*^ mice express the *Snord115* cluster ubiquitously, whereas the 5-Ht2c receptor mRNA is detectable in mouse brain areas but absent in spleen and heart^6,34^; we hence analyzed the presence of the most abundant Snord115 editing isoform in our mouse model. Reverse transcription followed by PCR analysis of A64 → I64 editing site for Snord115 uncovered the edited isoform only in the analyzed brain areas but not in spleen and heart of *PWScr*^*p*−*/m5*′*LoxP*^ mice (Supplementary Fig. 1, Supplementary Material and Methods). Our data might indicate that Snord115 and exon Vb of 5-Ht2cr pre-mRNA form an RNA-duplex that is target to ADAR-mediated A to I editing. This recalls RNA editing of the 5-Ht2cr pre-mRNA intron, which presumably requires the intramolecular duplex formation with exon Vb (Supplementary Fig. 1).

## Discussion

RNomics discovered a number of snoRNAs that not only direct site-specific 2′-O-methylation or pseudouridylation of rRNAs and snRNAs targets, but could possibly be involved in other cellular processes including those that underlie human diseases^10,35–37^. The imprinted Snord115 snoRNA gene cluster was originally identified almost two decades ago^16^. Similar to other “orphan” snoRNAs, Snord115 does not display significant base complementarities to rRNAs or snRNAs. Due to an 18 nucleotide base complementarity upstream from the snoRNA D-box the snoRNA was thought to participate in the regulation of pre-mRNA posttranscriptional processing^10,16^. In order to analyze the *in vivo* impact of this snoRNA on posttranscriptional processing of the serotonin receptor 5-Ht2cr pre-mRNA, we investigated the *PWScr*^*p*−*/m5*′*LoxP*^ mouse model, which—in contrast to wild type animals—expresses the Snord115 gene cluster in the choroid plexus; thus, it is co-localized with 5-Ht2cr transcripts at the cellular level. This mouse model enabled a novel *in vivo* investigation of potential snoRNA function. Previous *in vitro* and *ex-vivo* (i.e., transfection in non-neuronal cell culture or embryonic carcinoma cell line P19 neuronal differentiation) analyses suggested that Snord115 targets exon Vb of the 5-Ht2c receptor pre-mRNA and restricts access of the splicing machinery to an alternative 5′-donor splice site, which is located 13nt upstream from the snoRNA targeting region (Fig. 1A)^29,30,38^. This interaction has been reported to reduce the expression of the truncated 5-Ht2c-receptor isoform and increase the production of functional receptor mRNA^29,30,38^.

Recently, in order to mimic potential Snord115 functions, synthetic RNA oligonucleotides complementary to various regions of 5-Ht2cr pre-mRNA (aimed at increasing the ratio of the full-length splice variant) were designed^39^. Targeting different 5-Ht2cr pre-mRNA regions revealed diverse alternative splicing efficiency. Interestingly, antisense oligonucleotides complementary to the same exon Vb region as Snord115 had almost no impact on the outcome of alternative splicing^39^.

In addition, investigation of a PWS mouse model (*PWS-IC*+/−) that expresses barely detectable levels of Snord115 revealed contradicting results. Initially, it was suggested that the absence of Snord115 RNA does not influence alternative splicing of 5-Ht2cr pre-mRNA^31^. However, more recently, the same group reported an increased expression of the truncated receptor isoform in the hypothalamus of *PWS-IC*+/− mice^32^. The analysis of the “autistic” mouse model, which harbors a paternal duplication of the PWS-locus imprinted genes (*patDp/*+ mouse) also established a link between increased Snord115 expression and altered 5-Ht2c-receptor signaling^33^. Although Snord115 RNA expression levels were different in the aforementioned mouse models (~2 fold increase in *patDp/*+ mouse and almost no expression in *PWS-IC*+/−), both studies uncovered increased A to I editing of 5-Ht2cr pre-mRNA^31,33^. Apparently, the analysis of potentially functional roles of Snord115 in posttranscriptional processing of 5-Ht2c receptor pre-mRNA and receptor activity *via* the *PWS-IC*+/− and *patDp/*+ mouse models is associated with certain limitations. For instance, the paternal expression of genes encoded within the PWS locus is perturbed. Deregulation of *Necdin* (*Ndn*) or *Magel2* expression alters the serotonergic signaling in the corresponding knock-out models^40,41^. Hence, the investigation of *PWScr*^*p*−*/m5*′*LoxP*^ mice where the Snord115 gene cluster is expressed maternally and the activity of protein-coding genes within the PWS-locus remains unaltered, is certainly advantageous and avoids the aforementioned uncertainties.

In addition, there are certain technical limitations in detecting rare 5-Ht2cr mRNA editing isoforms. An accurate analysis of mRNA A to I editing requires sufficient sequencing depth^14^. Moreover, it was reported that different mouse genetic backgrounds exhibited significant variations in 5-HT2c-receptor mRNA editing^14^. Considering these pitfalls, comparative RNA deep sequencing analysis of choroid plexus samples from *PWScr*^*p*−*/m5*′*LoxP*^ and wild type siblings with the same genetic C57Bl/6 J background was performed. To avoid effects related to the estrous cycle, only male mice were selected for analysis. We did not observe any significant influence of Snord115 on alternative splicing of 5-Ht2cr pre-mRNA; i.e., there was no increase of the full-length receptor 5-Ht2cr mRNA level detectable within the choroid plexus of *PWScr*^*p*−*/m5*′*LoxP*^ mice compared to wild type animals. Our data established that irrespective of genotype, approximately 45% of all cDNAs represented the functional receptor mRNA, and ~55% the truncated isoform. Because *in vitro* and *ex vivo* data suggest that Snord115 targets exon Vb of the 5-Ht2c receptor pre-mRNA and increases the production of the full-length mRNA isoform, we can not completely exclude that the snoRNA itself is not sufficient for the regulation of alternative splicing *in vivo*, and that other factors might be missing in the choroid plexus.

The aforementioned 18 nucleotide region upstream from the D-box of Snord115 displays base complementarity to the 5-Ht2cr pre-mRNA^16,17,28^. In case of canonical C/D-box snoRNAs, the fifth nucleotide upstream from the D-box usually determines the site of 2′-O-methylation, which for the serotonin receptor pre-mRNA would correspond to the C-editing site. In agreement, it has been demonstrated that Snord115 guides 2′-O-methylation and regulates C-site editing for nucleolar-localized mRNA substrates; this implies the involvement of ADAR2 in nucleolar RNA editing^13^. Our analysis indicated that Snord115 potentially modulates RNA editing within the entire targeting region, as revealed by different isoform frequencies of edited 5-Ht2c receptor mRNAs for *PWScr*^*p*−*/m5*′*LoxP*^ and wild type samples. Interestingly, the edited A and B sites do not overlap with the Snord115 complementary region. Changes in RNA editing observed at A and B-sites suggest that Snord115 might compete with pre-mRNA intronic sequences disturbing the exon-intron duplex formation. Moreover, as ADAR1 is not known to be involved in nucleolar RNA editing, Snord115 pre-mRNA interaction most probably occurs in the nucleoplasm and stimulates formation of alternative pre-mRNA secondary structures^13^. Recent findings suggest that RNA editing also occurs co-transcriptionally and is possibly coupled to R-loops in mammalian cells^42^.

The analysis of the subcellular distribution of Snord115 ribonucleoprotein (RNP) complexes indicated that fractions of the snoRNA localize to the nucleoplasm^43^. Interestingly, here we show that even Snord115 itself is subject to low-level A to I editing in mouse brain. Moreover, editing was specifically detected at residues located within the Snord115 region, which supposedly is targeting exon Vb of 5-Ht2cr pre-mRNA (Supplementary Fig. 1). Analysis of Snord115 A64 → I64 editing in *PWScr*^*p*−*/m5*′*LoxP*^ mice revealed the edited isoform only in brain areas where *5-Ht2cr* is co-expressed with Snord115, but not in spleen and heart. We suggest that the double-stranded structure formed by Snord115 and exon Vb of 5-Ht2cr pre-mRNA is also subject to ADAR-mediated A to I editing of the snoRNA; i.e., similar to the putative intramolecular duplex formed by exon Vb and the downstream intron of 5-Ht2cr pre-mRNA (Supplementary Fig. 1)^13^. This might also explain differences in RNA editing for A and B compared to C and D sites in the presence of Snord115 RNA (Table 3). In contrast to C, E and D, the A and B editing sites do not overlap with the snoRNA-targeting region (Fig. 1A). As a consequence, RNA duplex structures, which are a necessary prerequisite for effective editing, cannot be established, resulting in the reduction of ADAR1-mediated editing. RNA editing in C, E and D sites might, however, proceed due to pre-mRNA/Snord115 interactions.

Collectively, our analysis of potential Snord115 involvements in the posttranscriptional processing of 5-Ht2c receptor pre-mRNA *in vivo* suggests that, ectopically expressed, the snoRNA does not control alternative splicing in choroid plexus, but might participate in the fine-tuning regulation of posttranscriptional editing. To the best of our knowledge, this is the first Snord115 gain-of-function analysis based on *in vivo* mouse models. However, it is not clear whether these small changes in A to I editing are of biological relevance. Generation and analysis of a Snord115 knock-out mouse model will be the next important step in the understanding of Snord115 – 5-Ht2cr functional interaction (if any).

## Materials and Methods

### Generation and genotyping of mice

Construction of the 5′-LoxP targeting cassette, generation of *PWScr*^*p*−*/m5*′*LoxP*^ mice and genotyping of analyzed mice were reported previously^20,34^.

### Northern blot analysis

Northern blot hybridization was performed as described earlier^27,44^. For each mouse brain area, total RNA samples were isolated and assayed from at least three different male animals of each genotype. In brief, 500 ng of each total RNA sample were separated on 8% (w/v) denaturing polyacrylamide gel (PAAG; 7 M urea, 1X TBE buffer) at 200 V for 90 min and transferred onto positively charged nylon membranes (BrightStar Plus, Ambion). Oligonucleotide probes for hybridizations were 5′-end labeled with γ-[^32^P]-ATP using T4 polynucleotide kinase (New England Biolabs) (For oligonucleotide sequences, see Supplementary Table 4). Membranes were pre-hybridized in 20 ml of 0.5 M sodium phosphate (pH 6.5 at 58 °C), 7% (w/v) sodium dodecyl sulfate (SDS) buffer at 56 °C for 30 minutes. Hybridizations were performed with 50 pmol of 5′-^32^P labeled oligonucleotides in pre-hybridization buffer at 56 °C overnight. Membranes were washed three times in 0.1 M sodium phosphate (pH 6.5), 1% (w/v) SDS containing buffer at 46 °C and exposed to MS-film (Kodak) overnight at −80 °C.

### Reverse-transcription quantitative real-time PCR (RT-qPCR)

Total RNA samples were DNase I (Roche) treated; concentration and purity were determined with a NanoDrop spectrophotometer ND-1000 (Thermo Scientific) and the absorbance at OD_260/280_. Total RNA integrity was examined on 8% (w/v) denaturing polyacrylamide gels (PAAG; 7 M urea, 1X TBE buffer). The presence of DNA contamination was assessed by PCR as described earlier^45^. First strand cDNA synthesis was performed using random hexamer primers and reverse transcriptase (Roche).

Total RNA (0.5 µg) was incubated with 1 µl of random hexamer primers (3 µg/µl), 1 µl of dNTP mix (25 mM each) and 9.5 µl of DEPC-treated water for 10 min at 65 °C. The reaction was cooled on ice for 2–3 min and briefly centrifuged. Then, 4 µl of first strand synthesis buffer (5X, containing 250 mM Tris-HCl (pH 8.5), 150 mM KCl, 40 mM MgCl_2_), 0.5 µl of ribolock RNAase inhibitor (40 U/µl, Fermentas) and 0.5 µl of (20 U/µl) of Transcriptor reverse transcriptase (Roche) were added and incubated for 60 min at 55 °C. The reaction was terminated by heat inactivation for 10 min at 85 °C. The reaction volume was increased to 50 µl using nuclease-free water stored at −20 °C. The resulting cDNA was diluted 1:10 and used in real-time PCR reactions.

All details on the primer sequences for RT-qPCR analysis in this study are provided in Supplementary Table 4. All qPCR reactions were performed in triplicate, and a total volume of 10 µl containing 2 µl of cDNA (~20 ng), 5 µl of 2X LightCycler 480 SYBR Green Master Mix (Roche) and 1 µM of each primer.

PCR amplification: 5 min initial denaturation step at 95 °C, with subsequent 45 cycles of 20 sec at 95 °C and 1 min at 60 °C. The reaction included single acquisition of fluorescent signals at 60 °C for each cycle and continuous acquisition from 50 °C to 97 °C at the end of the 45 cycles for melt-curve analysis. Quantification Cycle (Cq) values were calculated using Light-cycler 480 SW 1.5 software (Roche) and all data were further analyzed with Excel. Data analysis was performed using a geometric mean of ActB mRNA as reference genes. The fold change is represented as 2^−ddCq^ (Supplementary Table 2).

### Deep sequencing analysis of 5-Ht2cr pre-mRNA

Twelve cDNA libraries (6 per genotype) were generated. Choroid plexuses from 24 male mice (12 animals from each genotype) at postnatal day 21 were isolated; two choroid plexuses per library were pooled and subjected to total RNA extraction. cDNA synthesis was performed with total RNA samples 100 ng each using 200 pmol of 5ht2cRTrev oligonucleotide and 10 U of Transcriptor Reverse Transcriptase (Roche) in a 20 µl reaction as described above. Five µl aliquots of the RT-reactions were subjected to 10 cycles of PCR amplification with 5-Ht2cr exons IV and VI specific oligonucleotides: 5ht2cF and 5ht2cRTrev (Fig. 1A and Supplementary Table 4). PCR products were purified using High Pure PCR cleaning Micro Kit (Roche) and eluted in 20 µl of ddH2O. Ten µl of purified reactions were used for 15 cycles of PCR amplification to introduce PGM adapters with barcodes (Supplementary Table 4).

### Analysis of Serotonin receptor editing

All BAM files were converted to the tab delimited text format (SAM) by a batch call to the samtools view option (GEO submission number is GSE119419)^46^. Contigs mapped to our FASTA input sequences represent the two 5-Ht2cr variants: 1–209 and 5-Ht2cr-tr-sequence: 1–114, respectively. The resulting 12 SAM files were utilized to count frequencies of editing patterns (with expanded CIGAR information and high mapping quality) for all combinations of editing sites. To render the results comparable between samples, read counts were converted to RPKM expression values by normalizing the values of each sample to its own number of total mapped reads; RPKM = Reads/sequence length * (10^9/total mapped reads). Only isoforms with mean expression threshold levels of ≥10 RPKM in either wild type or *PWScr*^*p*−*/m5*′*LoxP*^ samples were analyzed. This established a matrix of RPKM values with 12 experiments in columns and 112 editing pattern in rows (including 1 non edited pattern).

### Statistics

All statistics were computed with functions and routines based on the statistical computing systems S+ and R (TIBCO® Spotfire S+ 8.1 for Windows, R version 3.3.2 (2016-10-31), Copyright (C) 2016)). R is the freely available implementation of the S-programming language and runs on UNIX, MAC and Windows systems (https://www.cran.r-project.org). Functions and R-routines are available on demand. For fast computation of large datasets, we vectorised standard statistical tests (Student’s t-test and correction for multiple testing) in our functions. P-values for t-tests of analyzed isoforms with mean expression levels of ≥10 RPKM were adjusted for multiple testing via the step-up procedure for correction of the ‘False Discovery Rate’ (FDR)^47^ (https://tools.carbocation.com/FDR).

For all manipulations on BAM files (BAM to SAM conversion, indexing, IDX-statistics), we used the 32-Bit Windows version of the freely available samtools (http://samtools.sourceforge.net)^46^.

### Mice

All mouse procedures were performed in compliance with the guidelines for the welfare of experimental animals issued by the Federal Government of Germany and approved by the State Agency for Nature, Environment and Consumer Protection North Rhine-Westphalia (Landesamt für Natur, Umwelt und Verbraucherschutz Nordrhein-Westfalen). Animals were kept in specific pathogen-free animal facilities. All breading conditions and the weaning of pups were accomplished as previously described^20,34^.

## Supplementary information

Supplementary information

Supplementary Table 3

## References

[1] Chagraoui A, Thibaut F, Skiba M, Thuillez C, Bourin M (2016). 5-HT2C receptors in psychiatric disorders: A review. Prog. Neuropsychopharmacology Biol. Psychiatry..

[2] De Deurwaerdere P, Lagiere M, Bosc M, Navailles S (2013). Multiple controls exerted by 5-HT2C receptors upon basal ganglia function: from physiology to pathophysiology. Exp. Brain Res..

[3] Palacios JM, Pazos A, Hoyer D (2017). A short history of the 5-HT2C receptor: from the choroid plexus to depression, obesity and addiction treatment. Psychopharmacology..

[4] Pritchard AL (2008). Role of 5HT 2A and 5HT 2C polymorphisms in behavioural and psychological symptoms of Alzheimer’s disease. Neurobiol. Aging..

[5] Abramowski D, Staufenbiel M (1995). Identification of the 5-hydroxytryptamine2C receptor as a 60-kDa N-glycosylated protein in choroid plexus and hippocampus. J. Neurochem..

[6] Yue F (2014). A comparative encyclopedia of DNA elements in the mouse genome. Nature..

[7] Burns CM (1997). Regulation of serotonin-2C receptor G-protein coupling by RNA editing. Nature..

[8] Herrick-Davis K, Grinde E, Niswender CM (1999). Serotonin 5-HT2C receptor RNA editing alters receptor basal activity: implications for serotonergic signal transduction. J. Neurochem..

[9] Price RD, Weiner DM, Chang MS, Sanders-Bush E (2001). RNA editing of the human serotonin 5-HT2C receptor alters receptor-mediated activation of G13 protein. J. Biol. Chem..

[10] Cavaille J (2017). Box C/D small nucleolar RNA genes and the Prader-Willi syndrome: a complex interplay. Wiley Interdiscip. Rev. RNA..

[11] Hartner JC (2004). Liver disintegration in the mouse embryo caused by deficiency in the RNA-editing enzyme ADAR1. J. Biol. Chem..

[12] Higuchi M (2000). Point mutation in an AMPA receptor gene rescues lethality in mice deficient in the RNA-editing enzyme ADAR2. Nature..

[13] Vitali P (2005). ADAR2-mediated editing of RNA substrates in the nucleolus is inhibited by C/D small nucleolar RNAs. J. Cell. Biol..

[14] Morabito MV (2010). High-throughput multiplexed transcript analysis yields enhanced resolution of 5-hydroxytryptamine 2C receptor mRNA editing profiles. Mol. Pharmacol..

[15] Olaghere da Silva UB (2010). Impact of RNA editing on functions of the serotonin 2C receptor *in vivo*. Front. Neurosci..

[16] Cavaille J (2000). Identification of brain-specific and imprinted small nucleolar RNA genes exhibiting an unusual genomic organization. Proc. Natl. Acad. Sci. USA.

[17] Nahkuri S, Taft RJ, Korbie DJ, Mattick JS (2008). Molecular evolution of the HBII-52 snoRNA cluster. J. Mol. Biol..

[18] Buiting K (2010). Prader-Willi syndrome and Angelman syndrome. Am. J. Med. Genet. C Semin. Med. Genet..

[19] Ding F (2008). SnoRNA Snord116 (Pwcr1/MBII-85) deletion causes growth deficiency and hyperphagia in mice. PLoS One..

[20] Skryabin BV (2007). Deletion of the MBII-85 snoRNA gene cluster in mice results in postnatal growth retardation. PLoS Genet..

[21] Bieth E (2015). Highly restricted deletion of the SNORD116 region is implicated in Prader-Willi Syndrome. Eur. J. Hum. Genet..

[22] de Smith AJ (2009). A deletion of the HBII-85 class of small nucleolar RNAs (snoRNAs) is associated with hyperphagia, obesity and hypogonadism. Hum. Mol. Genet..

[23] Sahoo T (2008). Prader-Willi phenotype caused by paternal deficiency for the HBII-85 C/D box small nucleolar RNA cluster. Nat. Genet..

[24] Runte M, Varon R, Horn D, Horsthemke B, Buiting K (2005). Exclusion of the C/D box snoRNA gene cluster HBII-52 from a major role in Prader-Willi syndrome. Hum. Genet..

[25] Cavaille J, Nicoloso M, Bachellerie JP (1996). Targeted ribose methylation of RNA *in vivo* directed by tailored antisense RNA guides. Nature..

[26] Watkins NJ, Bohnsack MT (2012). The box C/D and H/ACA snoRNPs: key players in the modification, processing and the dynamic folding of ribosomalRNA. Wiley Interdiscip. Rev. RNA..

[27] Mo D, Raabe CA, Reinhardt R, Brosius J, Rozhdestvensky TS (2013). Alternative processing as evolutionary mechanism for the origin of novel nonprotein coding RNAs. Genome Biol. Evol..

[28] Zhang YJ (2014). Rapid birth-and-death evolution of imprinted snoRNAs in the Prader-Willi syndrome locus: implications for neural development in Euarchontoglires. PLoS One..

[29] Kishore S, Stamm S (2006). The snoRNA HBII-52 regulates alternative splicing of the serotonin receptor 2C. Science..

[30] Bratkovic T, Modic M, Camargo Ortega G, Drukker M, Rogelj B (2018). Neuronal differentiation induces SNORD115 expression and is accompanied by post-transcriptional changes of serotonin receptor 2c mRNA. Sci. Rep..

[31] Doe CM (2009). Loss of the imprinted snoRNA mbii-52 leads to increased 5htr2c pre-RNA editing and altered 5HT2CR-mediated behaviour. Hum. Mol. Genet..

[32] Garfield AS (2016). Increased alternate splicing of Htr2c in a mouse model for Prader-Willi syndrome leads disruption of 5HT2C receptor mediated appetite. Mol. Brain..

[33] Nakatani J (2009). Abnormal behavior in a chromosome-engineered mouse model for human 15q11-13 duplication seen in autism. Cell..

[34] Rozhdestvensky TS (2016). Maternal transcription of non-protein coding RNAs from the PWS-critical region rescues growth retardation in mice. Sci. Rep..

[35] Huttenhofer A (2001). RNomics: an experimental approach that identifies 201 candidates for novel, small, non-messenger RNAs in mouse. EMBO J..

[36] Romano G, Veneziano D, Acunzo M, Croce CM (2017). Small non-coding RNA and cancer. Carcinogenesis..

[37] Stepanov GA (2015). Regulatory role of small nucleolar RNAs in human diseases. BioMed Res. Int..

[38] Kishore S (2010). The snoRNA MBII-52 (SNORD 115) is processed into smaller RNAs and regulates alternative splicing. Hum. Mol. Genet..

[39] Zhang Z (2016). Oligonucleotide-induced alternative splicing of serotonin 2C receptor reduces food intake. EMBO Mol. Med..

[40] Mercer RE (2009). Regionally reduced brain volume, altered serotonin neurochemistry, and abnormal behavior in mice null for the circadian rhythm output gene Magel2. Am. J. Med. Genet. B Neuropsychiatr. Genet..

[41] Zanella S (2008). Necdin plays a role in the serotonergic modulation of the mouse respiratory network: implication for Prader-Willi syndrome. J. Neurosci..

[42] Wang IX (2014). RNA-DNA differences are generated in human cells within seconds after RNA exits polymerase II. Cell Rep..

[43] Soeno Y (2010). Identification of novel ribonucleo-protein complexes from the brain-specific snoRNA MBII-52. RNA..

[44] Galiveti CR, Raabe CA, Konthur Z, Rozhdestvensky TS (2014). Differential regulation of non-protein coding RNAs from Prader-Willi Syndrome locus. Sci. Rep..

[45] Galiveti CR, Rozhdestvensky TS, Brosius J, Lehrach H, Konthur Z (2010). Application of housekeeping npcRNAs for quantitative expression analysis of human transcriptome by real-time PCR. RNA.

[46] Li H (2009). The Sequence Alignment/Map format and SAMtools. Bioinformatics..

[47] Benjamini Y, Hochberg Y (1995). Controlling the False Discovery Rate: A Practical and Powerful Approach to Multiple Testing. J. R. Statist. Soc. B..

