# Supplementary material for: Ectopic expression of Snord115 in choroid plexus interferes with editing but not splicing of 5-Ht2c receptor pre-mRNA in mice
Source: Sci Rep. 2019 Mar 12;9:4300. doi: 10.1038/s41598-019-39940-6 (PMC6414643; doi:10.1038/s41598-019-39940-6)
Supplement: Supplementary file 1 — Supplementary information [file 41598_2019_39940_MOESM1_ESM.pdf]

Supplementary information

Ectopic expression of Snord115 in choroid plexus interferes with editing but not splicing of 5-Ht2c receptor pre-mRNA in mice

Carsten A. Raabe[1,2,§], Reinhard Voss[3,§], Delf-Magnus Kummerfeld[4], Juergen Brosius[1,5], Chenna R. Galiveti[1], Anna Wolters[1], Jochen Seggewiss[6], Andreas Huge[7], Boris V. Skryabin[4*] and Timofey S. Rozhdestvensky[4*]

[1]Institute of Experimental Pathology (ZMBE), University of Muenster, Von-Esmarch-Str. 56, D-48149 Muenster, Germany; [2]Institute of Medical Biochemistry (ZMBE), University of Muenster, Von-Esmarch-Str. 56, D-48149 Muenster, Germany; [3]Laukamp 12, D-48161 Muenster, Germany; [4]Medical Faculty, Core Facility Transgenic animal and genetic engineering Models (TRAM), University of Muenster, Von-Esmarch-Str. 56, D-48149 Muenster, Germany; [5]Brandenburg Medical School (MHB), Fehrbellinerstr. 38, D-16816 Neuruppin; [6]Institute of Human Genetics, University Hospital Muenster, Vesaliusweg 12-14, D-48149 Muenster, Germany. [7]Medical Faculty, Core Facility Genomik, University of Muenster, Albert-Schweitzer-Campus 1, D3, Domagkstrasse 3, D-48149 Muenster Germany.

[*]Corresponding author: Timofey S. Rozhdestvensky: rozhdest@uni-muenster.de, correspondence also can be addressed to Boris V. Skryabin: skryabi@uni-muenster.de.

[§]The authors wish it to be known that, in their opinion, the first two authors should be regarded as joint First Authors.

Running title: Snord115 and 5-Ht2c receptor pre-mRNA processing

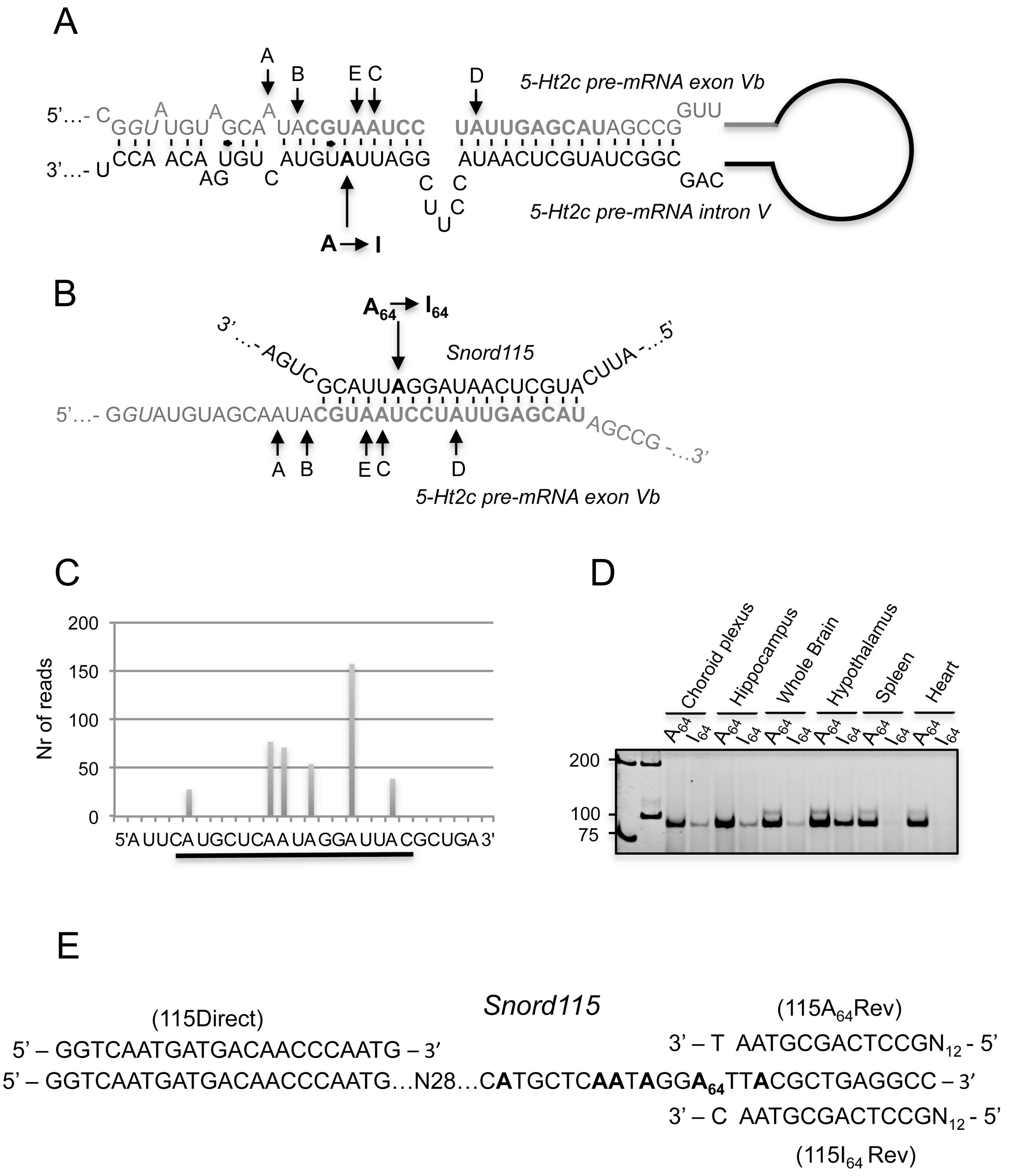

Supplementary Figure 1. Schematic representation of 5-Ht2cr pre-mRNA, exon Vb targeting by Snord115 and putative snoRNA edited isoforms.

A. Putative secondary structure formed between partial regions of exon –Vb (red) and intron –V (black) of 5-Ht2cr pre-mRNA. B. Putative base pairing of Snord115 with exon Vb of 5-HT2cr pre-mRNA. A, B. The alternative splice site (*GU*) of exon Vb and A to I editing sites in 5HT2c pre-mRNA (A-D) are indicated. A to I editing of 5Ht2cr pre-mRNA intron V and A64 of the Snord115 are depicted by black arrows, respectively. C. Analysis of ~10.000 Snord115 cDNA reads derived from a whole

brain mouse cDNA-library representing the small non-protein-coding transcriptome. Number of edited reads (y – coordinate) for the Snord115 guide region (x-coordinate) is shown. D. RT-PCR analysis of total RNA isolated from spleen, heart and different brain areas (as indicated above the blot) of $PWScr^{p-/m5'LoxP}$ mice (see Supplementary Material and Methods). PCR products were resolved on 8 % (w/v) polyacrylamide gel (PAAG (30:1); 1 X TBE buffer). Electrophoresis was performed at 120 V for 60 min in 1 X TBE buffer. A negative image of the ethidium bromide stained gel is shown. E. Snord115 and PCR primer sequences are displayed. Putatively edited nucleotides are indicated in bold.

Supplementary Material and Methods.

Total RNA samples were DNase I (Roche) treated. Reverse transcription was performed with RT115Rev (5'-CCTCTCTATGGGCAGTCGGTGAT<u>GCCTCAGCG</u> -3') and reverse transcriptase (Roche). Total RNA (~5µg; choroid plexus ~300 ng) was incubated with 1 µl of RT115Rev primer (100 pmole/µl) for 2 min at 85°C in 10 µl reaction volume. The reaction was cooled at room temperature for 3-5 min and briefly centrifuged. Then, 1 µl of dNTP mix (25 mM each), 4 µl of first strand synthesis buffer (5 X, containing 250 mM Tris-HCl (pH 8.5), 150 mM KCl, 40 mM $MgCl_2$), 0.5 µl of ribolock RNase inhibitor (40 U/µl, Fermentas) and 0.5 µl (20 U/µl) of Transcriptor reverse transcriptase (Roche) were added and incubated for 60 min at 55°C. The reaction was terminated by heat inactivation for 10 min at 85°C. Resulting cDNA was diluted 1:10 and used for PCR reactions. All PCR reactions were performed in triplicates, in a total reaction volume of 50 µl, containing 1 µl of 1:10 diluted cDNA (10 µl in case of choroid plexus cDNA), 400 µM of dNTP, 1 µM each primer and ~2,5 U of Taq polymerase. For the detection of A64 and I64 Snord116 isoforms two PCR master mixes were prepared. The A64 master mix contained forward primer (115Direct: 5'-GGTCAATGATGACAACCCAATG-3') and A64 specific reverse primer (115RevA64: 5'-GCAGTCGGTGATGCCTCAGCGTAAT-3'). The I64 detection master mix comprised of 115Direct primer and I64 specific reverse primer (115RevI64 5'-GCAGTCGGTGATGCCTCAGCGTAAC-3'). PCR amplification: 3 min initial denaturation step at 95°C, with subsequent 30 cycles of 30 sec at 95°C, 20 sec at 58°C and 30 sec at 72°C.

Supplementary Table 1. Read statistic of all 12 BAM files

Genotype	cDNA name	cDNA length	mapped reads	%	total mapped reads
LOXP.007	5-Ht2cr	209	1997730	41.37	4828960
LOXP.007	5-Ht2cr-tr	114	2831230	58.63	4828960
LOXP.008	5-Ht2cr	209	2033062	42.23	4813828
LOXP.008	5-Ht2cr-tr	114	2780766	57.77	4813828
LOXP.009	5-Ht2cr	209	3655972	48.86	7482758
LOXP.009	5-Ht2cr-tr	114	3826786	51.14	7482758
LOXP.010	5-Ht2cr	209	3747832	48.51	7726413
LOXP.010	5-Ht2cr-tr	114	3978581	51.49	7726413
LOXP.011	5-Ht2cr	209	3551429	46.24	7680953
LOXP.011	5-Ht2cr-tr	114	4129524	53.76	7680953
LOXP.012	5-Ht2cr	209	2669286	41.53	6427864
LOXP.012	5-Ht2cr-tr	114	3758578	58.47	6427864
WT.001	5-Ht2cr	209	3482201	43.19	8062141
WT.001	5-Ht2cr-tr	114	4579940	56.81	8062141
WT.002	5-Ht2cr	209	3844526	44.95	8553154
WT.002	5-Ht2cr-tr	114	4708628	55.05	8553154
WT.003	5-Ht2cr	209	3133636	46.23	6778386
WT.003	5-Ht2cr-tr	114	3644750	53.77	6778386
WT.004	5-Ht2cr	209	3161517	46.26	6834878
WT.004	5-Ht2cr-tr	114	3673361	53.74	6834878
WT.005	5-Ht2cr	209	755291	46.27	1632419
WT.005	5-Ht2cr-tr	114	877128	53.73	1632419
WT.006	5-Ht2cr	209	723863	46.44	1558622
WT.006	5-Ht2cr-tr	114	834759	53.56	1558622

Genotype: genotypes of investigated mice: LOXP - $PWScr^{p-/m5'LoxP}$ and WT - wild type mice. Numbers: 001-012 is the BAM files designation. *cDNA name/cDNA length*: reads represented by full-length 5-Ht2cr mRNA or truncated (alternatively-spliced) 5-Ht2cr-tr mRNA and its corresponding length. *Mapped reads*: number of reads for the 5-Ht2cr or 5-Ht2cr-tr transcripts. *%*: percent cDNA reads representing the functional and truncated receptor isoforms. *Total mapped reads*: value of mapped reads for the indicated BAM files.

Supplementary Table 2. Analysis of ADAR1 and ADAR2 RNA expression in total brain (A) and choroid plexus (B) samples of *PWScr*$^{p-/m5'LoxP}$ and wild type mice.

A

mice	mRNA	DCq	DCqStDev	DDCq	DDCq+ StDev	DDCq- StDev	2^-DDcq	2^-DDcq (+StDev)	2^-DDcq (-StDev)
Wt	ADAR1	7.08	0.19595	0	0.196	0.196	1	0.873	1.146
LoxP	ADAR1	6.95	0.14351	0.13	0.275	-0.012	0.91	0.827	1.009
Wt	ADAR2	8.32	0.19828	0	0.198	-0.198	1	0.872	1.147
LoxP	ADAR2	8.23	0.15943	0.084	0.244	-0.075	0.94	0.844	1.053

B

mice	mRNA	DCq	DCqStDev	DDCq	DDCq+ StDev	DDCq- StDev	2^-DDcq	2^-DDcq (+StDev)	2^-DDcq (-StDev)
Wt	ADAR1	5.31	0.09669	0	0.097	-0.097	1	0.935	1.069
LoxP	ADAR1	5.03	0.06279	0.28	0.343	0.217	0.824	0.789	0.86
Wt	ADAR2	10.04	0.17207	0	0.172	-0.172	1	0.888	1.127
LoxP	ADAR2	9.88	0.07577	0.16	0.236	0.084	0.895	0.849	0.943

Mice: each sample, was represented by three mouse brains (A) or six and four choroid plexuses (B) in case of wild type and *PWScr*$^{p-/m5'LoxP}$ mice, respectively. Mouse genotypes are indicated as Wt for wild type and LoxP for *PWScr*$^{p-/m5'LoxP}$ animals. (**A**, **B**) Each sample was analyzed in triplicate. *mRNA*: Adenosine deaminase acting on RNA 1 and 2 (ADAR1 and ADAR2, respectively) mRNAs. Columns: DCq is the average delta Cq values from technical and biological replicates; DCqStDev is the average of delta Cq standard deviations; DDCq: is the abbreviation for delta-delta Cq values.

Supplementary Table 4. Oligonucleotides.

DNA oligonucleotides for deep sequencing analysis of 5-Ht2cr pre-mRNA

Name	Sequence
m5HT2cF	gtctggatttcactagatgtgct
m5HT2cR	ctttcgtccctcagtccaatcac
trP1_5htR	CCTCTCTATGGGCAGTCGGTGATctttcgtccctcagtccaatcac
A5htb1F	CCATCTCATCCCTGCGTGTCTCCGACTCAGCTAAGGTAACg tctggatttcactagatgtgct
A5htb2F	CCATCTCATCCCTGCGTGTCTCCGACTCAGTAAGGAGAACg tctggatttcactagatgtgct
A5htb3F	CCATCTCATCCCTGCGTGTCTCCGACTCAGAAGAGGATTCg tctggatttcactagatgtgct
A5htb4F	CCATCTCATCCCTGCGTGTCTCCGACTCAGACCAAGATCgtc tggatttcactagatgtgct
A5htb5F	CCATCTCATCCCTGCGTGTCTCCGACTCAGCAGAAGGAAC gtctggatttcactagatgtgct
A5htb6F	CCATCTCATCCCTGCGTGTCTCCGACTCAGCTGCAAGTTCgt ctggatttcactagatgtgct
A5htb13F	CCATCTCATCCCTGCGTGTCTCCGACTCAGTCTAACGGACg tctggatttcactagatgtgct
A5htb14F	CCATCTCATCCCTGCGTGTCTCCGACTCAGTTGGAGTGTCgt ctggatttcactagatgtgct
A5htb15F	CCATCTCATCCCTGCGTGTCTCCGACTCAGTCTAGAGGTCgt ctggatttcactagatgtgct
A5htb16F	CCATCTCATCCCTGCGTGTCTCCGACTCAGTCTGGATGACgt ctggatttcactagatgtgct
A5htb17F	CCATCTCATCCCTGCGTGTCTCCGACTCAGTCTATTCGTCgt ctggatttcactagatgtgct
A5htb18F	CCATCTCATCCCTGCGTGTCTCCGACTCAGAGGCAATTGCg tctggatttcactagatgtgct

DNA oligonucleotides for RT-qPCR analysis

Name	Sequence
mActbF	AAGGCCAACCGTGAAAAGAT
mActbR	GTGGTACGACCAGAGGCATAC
mADAR1F1	TCCTGCAGTGACAAGATAGCA
mADAR1R1	GGTTCCACGAAAATGCTGAG
mADAR2F1	AAACAGGCCTCTCCTTAGTGG
mADAR2R1	GCACTGAAGTGGGGAGACTT

DNA oligonucleotides for northern blot hybridizations

Name	Sequence
Snord115R	CAGCGTAATCCTATTGAGCATGAAT
Snord116R	GTTCCGATGAGAGTGGCGGTACAGAGT
Snora35R	TGTACCCACCTGCATGCGGGAGTAGCCCAC
